# Biomedical Courses Should Also Be Designed for Dental Students: The Perceptions of Dental Students

**DOI:** 10.3390/dj9080096

**Published:** 2021-08-16

**Authors:** Fanny Mussalo, Terhi Karaharju-Suvanto, Päivi Mäntylä, Eeva Pyörälä

**Affiliations:** 1 Department of Oral and Maxillofacial Diseases, University of Helsinki and Helsinki University Hospital, 00014 Helsinki, Finland; terhi.karaharju-suvanto@helsinki.fi; 2 Institute of Dentistry, University of Eastern Finland, 70211 Kuopio, Finland; paivi.mantyla@uef.fi; 3 Oral and Maxillofacial Clinic, Kuopio University Hospital, 70029 Kuopio, Finland; 4 Center for University Teaching and Learning, University of Helsinki, 00014 Helsinki, Finland; Eeva.pyorala@helsinki.fi

**Keywords:** dental students, biomedical sciences, vertical integration, curriculum reform, interprofessional learning

## Abstract

Introduction: It can be challenging integrating biomedical sciences into dentistry programs. The aim was to examine students’ perceptions of how joint biomedical courses with medical students and courses tailored for dental students supported their clinical studies. Materials and methods: The target group was clinical phase dental students. Cross-sectional survey data were collected using a questionnaire, which consisted of questions covering biomedical and clinical study content and learning methods. Results: A total of 110 (82%) students completed the survey. Students had difficulty recognising the relevance of joint biomedical courses for clinical work, but when the link was clear, their interest in the content increased. The closer the respondents were to graduation, the less relevance they expressed the biomedical sciences had. Almost all students (95%) wanted more dental content for the early study years. Discussion: The student perspective provides valuable information for the development of biomedical courses. Students should be offered customised courses that include dental content and perspectives on clinical work, whenever suitable to the didactic content of the basic science course. Our study shows that the dental perspective needs greater integration with the biomedical content. This also supports interprofessional learning and appreciation for the other field’s contribution to human health.

## 1. Introduction

Globally, in most dental education units, dentistry programs are generally started with so-called preclinical courses lasting one to two years, focusing on biomedical content. Often these are carried out as shared learning for dental and medical students, and with medical students representing the majority, much of the material and assignments are primarily designed for them [1,2]. Most dental educators agree that biomedical knowledge forms an important part of the dental curriculum and provides enough life sciences evidence for the clinical practice in dentistry [3,4,5]. Studying biomedical sciences jointly with medical students requires attentive curriculum planning and good communication between teaching faculties, so that learning is effective for both groups, and marginalisation of dental students is reduced [6].

Recent research has shown that dental students were overwhelmed [7,8] and demotivated [6,9] by the abundance of biomedical study content, and emphasising the dental context could engage and enhance the dental curriculum [10]. Several studies have pointed out that dental students had difficulty recognising the link between biomedical courses and dental clinical practice [11,12]. In terms of motivation, it would be important to specify the relevance of the biomedical courses for the students [7,10]. In addition, the design of dental content influenced students’ motivation, effectiveness and stress levels [13]. Interactive, small-group case-based activities effectively increased students’ comfort levels and readiness to initiate clinical procedures [14]. First-year dental students have been found to be more interested in clinical than biomedical topics [15]. However, an important finding is that students who studied jointly with medical students expressed that they were better qualified to treat dental patients with medical conditions [12].

Interprofessional learning aims to bring together different professionals to learn with, from and about one another to collaborate more effectively in the delivery of safe, high-quality care for patients [16]. Health education has actively sought to promote interprofessional learning for more than two decades. A systematic review [17] revealed that learners responded positively to this type of learning and their attitudes and perceptions, as well as their collaboration and skills, improved in interprofessional learning. Less evidence was found for the effects on behaviour and patient care. Interprofessional learning should be tailored so that its contents and practices are relevant and of interest to all learners, and all students participating should feel equally valued [17]. In many dental education units, medical and dental students learn biomedicine together, but there is still little evidence of the positive effects of interprofessional learning in this style of teaching.

In 2020, the Association for Dental Education in Europe (ADEE) published a new consensus report on biomedical study content in European dental degree programs [3]. The consensus group claimed that progress had been made both in integrating courses horizontally between the disciplines and vertically over the successive study years. The report suggested that the so-called “2 + 3 model”, in which the degrees were divided into two preclinical and three clinical years, was no longer generally recommended. Instead, it was increasingly typical for degree programs to address the clinical topics in the early years and correspondingly bring in the elements of basic research into the clinical years. Involving dental students’ views is valuable in evaluating the success of integration. Of particular interest are their views on whether biomedical content has supported their clinical learning and the requirements of clinical work, and thus meets the prerequisites of a high-quality dental curriculum.

Students are important stakeholders in developing dental education [18]. They provide feedback on the quality of their education in course evaluations as well as in post-graduation surveys evaluating the working-life relevance of what they had studied. They also identify both formal and informal learning requirements, that is, what is written in the syllabus and what they are actually required to pass the courses.

At the University of Helsinki, the dental programs are conducted within the Faculty of Medicine, in which there are degree programs for both medical and dental students. The first two years of biomedical sciences are common for both medical and dental students. Many dental degree programs face the same challenge of how to integrate biomedical sciences into the dental curriculum. This study explores this issue from the perspective of clinical phase dental students. The aim of the study was to examine the early-stage biomedical courses from the students’ perspective and provide research-based recommendation for revising a dental curriculum. We hope to shed light on the aim by answering the following research questions:

(1) How did dental students in the clinical phase evaluate the relevance of the joint biomedical courses with medical students and the separate preclinical courses designed for dental students?
(2) How did the evaluation of preclinical courses vary between the third-, fourth- and fifth-year dental students?
(3) What learning content (dental theoretical disciplines and elements) did the dental students propose should be added to the first two years of study?

## 2. Materials and Methods

### 2.1. Study Content

In 2000, a major curriculum reform was implemented in the dental and medical education at the University of Helsinki. The curriculum introduced continued as it was until 2016, largely based on the educational approaches adopted at that time. Before this reform, the first two years consisted of a significant proportion of lectures. Most of these lectures were replaced by problem-based learning (PBL) tutorials in groups of seven or eight medical students and one or two dental students. The key learning principles of PBL are that learning is based on real-life problems, and student learning is constructive, self-directed, collaborative and context-based [19]. A well-designed PBL promotes scientific attitude and offers students the opportunity to learn clinical topics from the beginning of their education [20]. However, in PBL tutorials at the University of Helsinki, there were no cases designed for dental students, and aspects of oral health were not included in the learning tasks or materials. Biomedical study courses were built around the physiology and anatomy of organ systems, which was a common way to teach these contents around the globe [1]. Basic science subjects were taught in entities within an integrative strategy. Other learning methods used were lectures, seminars, demonstrations and dissections. Dental and medical students attended the same lectures and sitting exams and were assessed in the same way. Thus, the 2000 curriculum reform was done in part at the expense of dental students. In 2016, when the data for this study were collected, the faculty members were in a situation in which they had sufficient experience of the challenges of the 2000 curriculum and were ready to design and implement a new curriculum.

Most of the professional dental content and clinical skills were taught during an intensive, stressful [21] three-year clinical phase at the dental department and the student clinic. The main learning methods used in these courses were lectures, self-directive learning, digital learning, clinical work under supervision, skills laboratory, peer-to-peer practices and procedures, patient care practice in pairs and comprehensive longitudinal patient care. The detailed content of the dental curriculum is presented in Appendix A.

### 2.2. Study Design, Participants and Questionnaire

The questionnaire (Appendix B) was addressed to the 134 clinical phase dental students at the University of Helsinki in April 2016. Data were collected among third-, fourth- and fifth-year dental students after lectures with a paper questionnaire. It consisted of multiple-choice and open-ended questions on nine different sections covering preclinical and clinical courses, curriculum content, learning outcomes and methods used for learning and assessment.

In this study, we analysed two of the nine sections of the questionnaire. We explored the items in which students were asked to evaluate how the preclinical courses laid a foundation for the dental clinical courses. Students were asked to rate the courses on a 5-point Likert scale. In addition, we analysed the responses to a question in which students were asked for suggestions on what topics should be added to the preclinical phase. Data from the other sections of the survey were used in other research and in the development of the dental curriculum.

### 2.3. Ethics

The research was carried out in accordance with the guidelines of the Declaration of Helsinki and the Finnish National Advisory Board on Research Ethics. Students were informed by email about the study prior to data collection. In the beginning of the questionnaire, the students were informed about the aims of the study, the contact information of the researchers and that answering was voluntary and anonymous (Appendix B). In the end of the questionnaire, students were asked to express their informed consent to participate in the study. The responses were collected and analysed anonymously, and the confidentiality was guaranteed throughout the process.

### 2.4. Statistical Analysis

SPSS 27.0 (IBM Corporation) was used in the analysis. The nonparametric Kruskal–Wallis one-way analysis of variance was used to test the statistical significance of differences between the categorical variables. Participants were clustered into groups based on their demographic background variables (age, gender, academic study year, previous studies). The Kruskal–Wallis test qualified for the five-level psychometric scale assuming that the variables were ultimately continuous. The cut-off *p*-value used was 0.05. To measure the reliability and internal consistency of the Likert scale used, the Cronbach’s alpha was calculated. A value for Cronbach alpha over 0.9 indicates a very good level of reliability.

## 3. Results

A total of 110 (82%) students completed the questionnaire. From the third study year 37 students (80%) responded, from the fourth study year 40 (77%) responded, and from the fifth study year 33 (77%) students responded. All respondents answered the multiple-choice questions in which they evaluated study-related aspects, and 62% answered open-ended questions.

### 3.1. Participant Background Information

The background data of the respondents are presented in Table 1: 71% were women and 29% men. Three-quarters of the respondents were under 30 years of age, and a quarter (27%) of them were older. Sixty percent of respondents had previous academic studies, and 40% had an academic degree.

### 3.2. Relevance of the Preclinical Studies

Students were asked to rate each preclinical course in the first two study years on how it laid the foundation for clinical dental education. The Cronbach’s alpha for the evaluations on the biomedical study courses was 0.900 and 0.942 for the study courses tailored for dental students.

Of the joint biomedical study courses with medical students (Table 2), the best evaluation from all respondents (n = 110) was given to the course covering pharmacology of antibiotics and protection against microbes with the mean (M) of 3.7 and standard deviation (SD) of 0.9. The lowest evaluation was given to molecular biology (M = 2.5, SD = 1.1) and the course covering endocrinology and the human reproductive system (M = 2.6, SD = 1.0). In most courses, the evaluations remained relatively similar, with the M ranging from 2.7 to 3.4.

Of the study courses tailored specifically for dental students (Table 3), the best evaluation from all respondents (N = 110) was given to the course that covers the scope of basic level information on clinical dental disciplines (M = 4.1, SD = 0.8), and the lowest evaluations were given to the courses covering professionalism (M = 2.9, SD = 1.0 and M = 3.1, SD = 1.0). Most of the courses received relatively similar evaluations, with the M ranging between 3.3 (SD = 1.0) and 3.7 (SD = 0.9).

Courses tailored specifically for dental students (M = 3.5) received somewhat higher ratings from respondents than joint biomedical courses for medical and dental students (M = 3.1).

### 3.3. Evaluation of Preclinical Courses by Academic Year

Respondents were gathered into groups based on their academic study year to find out whether there was a difference on how they evaluated the study courses.

Third-year students who were at the beginning of their clinical phase rated 79% of the biomedical study courses higher than the fourth- and fifth-year students. The result was statistically significant in half (53%) of the courses. The study courses for which the evaluations were statistically significant covered the following subjects (*p*-value): neurobiology (<0.001), the cardiovascular system (<0.001), pharmacology of antibiotics and protection against microbes (0.007), the respiratory system (0.033) and the digestive system and nutrition (<0.001).

### 3.4. Adding Dental Content to the Preclinical Phase

Participants were able to suggest which dental theoretical subjects and dental elements they would like to add to the first two study years.

**Figure 1:**
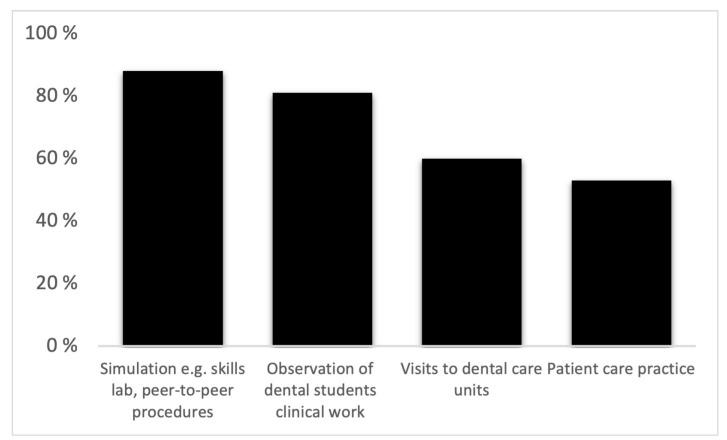
Elements proposed for the preclinical study phase of the dental curriculum (n = 110).

**Figure 2:**
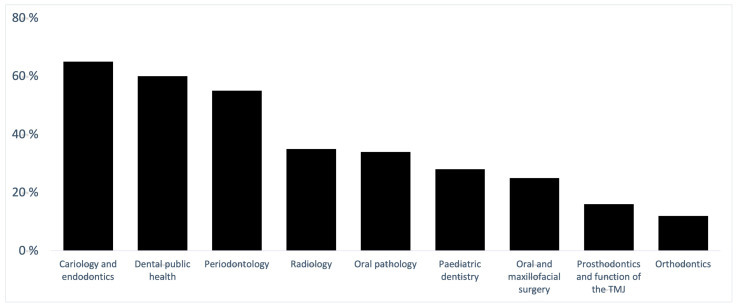
Dental theoretical subjects suggested for the preclinical study phase (n = 110).

Almost all students (95%) wanted more dental content, both theoretical and clinical in the first two study years. Most students wanted simulations, e.g., skills lab and peer-to-peer procedures (88%), observation of clinical work of dental students (81%) and visits to dental care units (60%). Students also wanted the following theoretical content for the early study years: cariology and endodontics (65%), dental public health (60%) and periodontology (55%). The elements and dental theoretical subjects that students wanted to add to the first two study years are presented in Figure 1 and Figure 2. In the open-ended answers, students wished that the curriculum would include, among other things, observation of a specialised dentist, practise of patients’ self-care with actors and work as a dental assistant.

## 4. Discussion

Dental units see biomedical study content as an important part of the degree program, but they face challenges in providing these courses in a way that interests and motivates dental students [3,4,5]. This is particularly the case for education units with both dental and medical degree programs, in which these studies are conducted mainly as joint courses that are primarily designed for medical students [1,2,10,11,12]. Research has found that in this type of dental unit, coordination between the basic sciences and dental subjects has been deficient [22].

This study was conducted in a university in which both medical and dental students are trained, and where dental students are clearly the minority. The aim of the study was to examine dental students’ perceptions of how joint biomedical courses and courses specifically tailored for dental students supported their clinical phase learning, and what they suggested should be added to their first few study years. The main findings of the study were as follows: (1) Dental students wanted more tailor-made dental courses in the first years of study. (2) The dental perspective should be integrated into joint courses with medical students. (3) The closer the respondents (third-, fourth- and fifth-year dental students) were to graduation, the less important they considered biomedical topics.

Research on dental education has shown that biomedical courses form an extensive part of the degree program [10]. Consistent with previous studies, we observed that students had difficulty recognising the relevance of joint biomedical courses for clinical work in dentistry [11,12], but when the link was clear, it increased their interest in the course content [7,8,10]. For example, courses that included pharmacology of antibiotics were graded more highly than courses covering embryology, molecular biology and genitals.

Studies have shown that dental students have felt themselves marginalised in the joint courses [7,8,9,21]. Our study demonstrated lost opportunities for interprofessional learning in dental education. The medical and dental students were learning biomedical sciences side by side for two years, but they were not learning with and from one another [16]. For joint biomedical courses to be mutually inspiring, they should be tailored so that course content includes both medical and dental topics and supports the active participation of both groups. Furthermore, all students should feel equally valued [17]. Without adequate planning, PBL alone did not guarantee students this opportunity. We need more research on how common biomedical sciences could be taught so that interprofessional learning would have positive outcomes. Designing courses of this type requires careful review of course evaluations and collaboration between biomedical, dental and medical teachers and students to make the study contents meaningful for both student groups. Furthermore, well-planned and well-timed dental courses during the preclinical phase support and maintain students’ motivation.

ADEE [23] suggests enhancing vertical integration, that is, including dental clinical material in the early years of study and adding biomedical subjects to clinical courses [3]. The results of our study were in line with ADEE suggestions and a previous study [15] which showed that students appreciated studies tailored specifically for dental students and agreed that more theoretical and practical content in dentistry could be included in the first two years of study in the curriculum. Students in this study proposed simulations, observation of dental students’ clinical work, visits to dental units and theoretical content in cariology and endodontics, dental public health and periodontology. However, organising courses tailored specifically for dental students might be challenging for small dental education units.

Even though students were not able to see the connection between the biomedical learning content and their future work, they still saw the usefulness [12]. Furthermore, according to one study, dental students preferred learning basic sciences together with medical students [24]. Prevention and treatment of oral diseases requires dental practitioners to have adequate theoretical and clinical competence. Studies have increasingly shown an association between oral health and medical conditions [25,26]. To understand these processes, it is essential for dentists to understand the basic principles of biomedicine. For example, a solid theoretical knowledge of endocrinology is needed in the treatment of patients with diabetes [27], a medical condition that affects almost 8.5% of the world’s population [28]. The emphasis on dental aspects in joint courses with medical students supports interprofessional learning and reminds of the importance of oral health for future physicians. If actively pursued by the faculty members, common learning of basic sciences develops connections and appreciation for the other field’s contribution to human health.

The objective of a university degree is not only to prepare students for clinical work but also to provide them with competence for lifelong learning, scientific thinking and a possible career as a researcher. This is a viewpoint that students might not consider whilst evaluating their studies. Undergraduate students’ views on their own education may not always provide the best course of action for designing an effective curriculum, and the perceptions of graduated dentists working in patient care and in research groups would complement the results of this study. Dentistry is a field in which research-based knowledge is growing rapidly as treatment techniques and practices evolve. The academic dental curriculum calls for a balance between the theoretical and clinical content taught.

In our study, we found that students studying in their last two years rated biomedical courses the lowest. Similar observations have been made in a recent study, in which undergraduate dental students found biomedical courses in some way relevant to their degree, but graduated dentists found them important only if they were heading for a scientific career [10]. This result supports the earlier observation that the more clinical work experience respondents had, the less relevant biomedical content seemed to be to them.

### Strengths and Weaknesses of the Study

Even though the response rate expressed as percentages was high, including almost all clinical phase students at the time when the data were collected, the number of respondents was relatively small due to the small annual intake of dental students. In addition, our study analyses the situation in one dental education unit, and the results as such cannot be generalised to other units. However, we have described the content of our study, and the course structure is provided in Appendix A. Therefore, we assume that the units in which dental education is conducted in a relatively similar way can benefit from the results of our study.

It would have been interesting to collect data from the first- and second-year students and compare the results to the third-, fourth- and fifth-year students’ evaluations. However, we thought it would be difficult for them to assess the clinical relevance of biomedical sciences. The collection of data over several years would have strengthened our results. The curriculum was reformed after the data were collected, and therefore the study could not be repeated as such.

## 5. Conclusions

Students’ perspectives on the basic biomedical sciences provide important information for developing the dental curriculum. Firstly, dental students should have study content designed for them specifically right from the start of their education. Secondly, the dental viewpoint should be incorporated into the joint courses with medical students and interprofessional learning promoted. Thirdly, as interest in biomedical sciences declined as studies progressed, these topics should be meaningfully integrated into the clinical phase of the undergraduate degree of dentistry.

## Figures and Tables

**Table 1:** Background information on the respondents (n = 110).

Age	N	%
Over 30	30	27
Under 30	78	71
Not mentioned	2	2
**Gender**		
Female	78	71
Male	26	24
Not mentioned	6	5
**Study year**		
3rd	37	34
4th	40	36
5th	33	30
**Previous studies**		
Academic degree	28	25
Academic studies	39	35
Healthcare studies	4	4
Other previous studies	1	1
No previous studies	37	34

**Table 2:** Students’ responses to the question asking them to evaluate how common biomedical courses had laid the foundation for dental clinical courses. Mean values (M) and standard deviations (SD) of the course evaluations.

Study Course	3rd–5th N = 110 M (SD)	3rd N = 37 M (SD)	4th N = 40 M (SD)	5th N = 33 M (SD)	*p*-Value
Dealing with an emergency or crisis situation	3.3 (0.9)	3.6 (1.0)	3.2 (0.8)	3.2 (0.9)	0.070
Medical biochemistry and pharmacology	3.3 (0.9)	3.5 (1.1)	3.2 (0.9)	3.3 (0.8)	0.380
Cellular biology and basic tissues	3.2 (0.9)	3.3 (0.8)	3.2 (0.9)	3.0 (1.0)	0.141
Metabolism and its regulation	2.8 (0.9)	2.8 (0.9)	2.7 (0.9)	2.9 (1.0)	0.435
Molecular biology	2.5 (1.1)	2.5 (1.0)	2.5 (0.9)	2.5 (1.1)	0.939
Embryology	2.7 (0.9)	2.7 (0.9)	2.6 (0.9)	2.7 (1.0)	0.739
Neurobiology	3.2 (0.8)	3.5 (0.7)	3.2 (0.7)	2.8 (0.9)	<0.001 *
Physiology and anatomy of the musculoskeletal system	3.2 (1.0)	3.5 (0.9)	3.2 (1.0)	3.0 (0.9)	0.108
Heart, circulatory system and kidney	3.4 (0.9)	4.0 (0.7)	3.2 (0.8)	3.1 (0.9)	<0.001 *
The surrounding environment, body’s defence and protection	3.7 (0.9)	4.1 (0.8)	3.7 (1.0)	3.4 (0.9)	0.007 *
The respiratory system	3.0 (0.9)	3.3 (0.9)	2.9 (0.9)	2.8 (0.9)	0.033 *
The digestive system and nutrition	3.3 (1.0)	3.8 (0.9)	3.2 (0.8)	2.9 (0.9)	<0.001 *
Endocrinology and genitals	2.6 (1.0)	2.8 (0.9)	2.6 (1.0)	2.5 (1.0)	0.432
Study courses overall mean	3.1	3.4	3.1	3.0	

* The differences between the evaluations between the groups is statistically significant. Cronbach α for the scale used 0.900. Scale: 1 = Not at all, 2 = Slightly, 3 = Moderately, 4 = Well, 5 = Very well.

**Table 3:** Students’ responses to the question asking them to evaluate how the courses tailored specifically for the dental students had laid a foundation for the clinical courses in dentistry. Mean values (M) and standard deviations (SD) of the course evaluations.

Study Course	3rd–5th N = 110 M (SD)	3rd N = 37 M (SD)	4th N = 40 M (SD)	5th N = 33 M (SD)	*p*-Value
Professionalism—study course 1	2.9 (1.0)	3.2 (0.9)	2.7 (0.9)	2.9 (1.1)	0.060
Interaction with a paediatric patient	3.3 (1.0)	3.7 (0.9)	3.2 (0.9)	3.1 (1.1)	0.038 *
Professionalism—study course 2	3.1 (1.0)	3.5 (0.9)	2.9 (0.9)	3.0 (1.1)	0.052
Paediatric dentistry	3.7 (0.9)	3.8 (0.9)	3.8 (0.9)	3.6 (0.9)	0.595
Face, mouth and teeth	4.1 (0.8)	4.6 (0.5)	4.0 (0.9)	3.8 (0.8)	<0.001 *
Feel the clinic	3.7 (1.2)	4.0 (1.2)	3.1 (1.2)	3.9 (1.0)	0.034 *
Study courses overall mean	3.5	4.0	3.8	3.7	

* The differences between the evaluations between the groups is statistically significant. Cronbach α for the scale used 0.942. Scale: 1 = Not at all, 2 = Slightly, 3 = Moderately, 4 = Well, 5 = Very well.

## Data Availability

The data presented in this study are available on request from the corresponding author.

## Appendix A

**Table A1:** Annex 1. Curriculum content and the number of ECTS (European Credit Transfer and Accumulation System) credits per study year at the University of Helsinki (for students who started their studies before the year 2015).

Study Year	Common Studies with the Medical Students	ECTS	Dental Studies	ECTS
1st	Dealing with an emergency or crisis situation, Medical biochemistry and pharmacology, Cellular biology and basic tissues, Metabolism and its regulation, Molecular biology, Embryology and part of Neurobiology	43/55 ECTS	Professionalism, Interaction with a paediatric patient, Face, mouth and teeth (basic anatomy and physiology), Working as a dental assistant for an upper year student	12/55 ECTS
2nd	Neurobiology continues, Physiology and anatomy of the musculoskeletal system, Heart, circulatory system and kidney, The surrounding environment, Body’s defence and protection, The respiratory system, The digestive system and nutrition and Endocrinology and genitals	45/53 ECTS	Paediatric dentistry, Dental public health, Medical microbiology	8/53 ECTS
3rd			20% of the studies consist of clinical work (patient care in pairs). Basic theoretical and clinical courses on periodontology, orthodontics, head and neck surgery, radiology, function of the TMJ, prosthodontics, cariology and endodontics.	58 ECTS
4th			40% of the studies consist of clinical work (procedures for patients, comprehensive longitudinal patient care). Theoretical courses of oral pathology, paediatric dentistry, prosthetics. Advanced courses on periodontology, head and neck surgery, orthodontics, radiology, orthodontics.	62 ECTS
5th			40% of the studies consist of clinical work (comprehensive longitudinal patient care). Refresher courses on all subjects and final written examinations	49 ECTS
6th			Six months of vocational training as a dental practitioner in a health centre	30 ECTS
	Language studies 6 ECTS Optional studies 10 ECTS	21/41 ECTS	Thesis 20 ECTS	41 ECTS

## Appendix B

Annex 2. Questionnaire designed for the study. How has the teaching of the medical faculty supported my growth into becoming a dentist?

________________________________________________________________________________________________________________

Study year	☐3rd	☐4th	☐5th
Age	☐Under 30	☐Over 30	
Gender	☐Female	☐Male	
Previous studies (other than dental studies)			☐University degree ☐University studies
			☐Health care studies
			☐Other previous studies
			Which studies ____________________________

**Table A2:** I Preclinical studies. Evaluate how the study courses of the first two study years have formed basis for the clinical phase studies. Circle the option “Not applicable” if you have not attended to the study course.

	Not at All	Slightly	Moderately	Well	Very Well	Not Applicable
Dealing with an emergency or crisis situation	1	2	3	4	5	0
Medical biochemistry and pharmacology	1	2	3	4	5	0
Cellular biology and basic tissues	1	2	3	4	5	0
Metabolism and its regulation	1	2	3	4	5	0
Molecular biology	1	2	3	4	5	0
Embryology	1	2	3	4	5	0
Neurobiology	1	2	3	4	5	0
Physiology and anatomy of the musculoskeletal system	1	2	3	4	5	0
Heart, circulatory system and kidney	1	2	3	4	5	0
The surrounding environment, body’s defence and protection	1	2	3	4	5	0
The respiratory system	1	2	3	4	5	0
The digestive system and nutrition	1	2	3	4	5	0
Endocrinology and genitals	1	2	3	4	5	0

**Table A3:** II Studies designed specifically for dental students during the first two study years. Evaluate how the studies designed specifically for the dental students during the first two study years have formed basis for the clinical phase studies. Circle the option “Not applicable” if you have not attended to the study course.

	Not at All	Slightly	Moderately	Well	Very Well	Not Applicable
Professionalism—study course 1	1	2	3	4	5	0
Interaction with a paediatric patient	1	2	3	4	5	0
Professionalism—study course 2	1	2	3	4	5	0
Paediatric dentistry	1	2	3	4	5	0
Face, mouth and teeth	1	2	3	4	5	0
Feel the clinic	1	2	3	4	5	0

What kind of dental studies would you suggest to add to the 1st and/or 2nd dental study year? Check your preferred options and also check the option 1st/2nd if the studies should be targeted specifically to either study year.

☐Simulation, i.e., skills lab, demonstrations	☐1st	☐2nd
☐Patient care	☐1st	☐2nd
☐Observation of clinical work of dental students	☐1st	☐2nd
☐Visits to dental care units	☐1st	☐2nd
☐Professionalism –studies	☐1st	☐2nd
☐Other, what?_____________________________________________________________________________		
Dental theoretical subjects		
☐Radiology	☐1st	☐2nd
☐Oral and maxillofacial surgery	☐1st	☐2nd
☐Oral pathology	☐1st	☐2nd
☐Periodontology	☐1st	☐2nd
☐Cariology and endodontics	☐1st	☐2nd
☐Paediatric dentistry	☐1st	☐2nd
☐Prosthodontics and the function of TMJ	☐1st	☐2nd
☐Orthodontics	☐1st	☐2nd
☐Dental public health	☐1st	☐2nd

**Table A4:** III Learning methods. Evaluate how different learning methods support your learning. Circle the option “Not applicable” if you have not participated in the learning method.

	Not at All Useful	Slightly Useful	Moderately Useful	Very Useful	Extremely Useful	Not Applicable
Lectures	1	2	3	4	5	0
Small group teaching	1	2	3	4	5	0
PBL	1	2	3	4	5	0
Seminars	1	2	3	4	5	0
Peer-to-peer procedures	1	2	3	4	5	0
Skills lab	1	2	3	4	5	0
Demonstrations	1	2	3	4	5	0
Patient care practice in pairs	1	2	3	4	5	0
Before-class learning activities	1	2	3	4	5	0
Digital learning						
Digital applications	1	2	3	4	5	0
Videos	1	2	3	4	5	0
Thesis	1	2	3	4	5	0
Comprehensive longitudinal patient care	1	2	3	4	5	0
Procedures for patients	1	2	3	4	5	0
Self-directive learning	1	2	3	4	5	0

Which learning method(s) in the third year best supported the transition to clinical care practice (working with patients)?

______________________________________________________________________________________________________________________________________________________________________________________________________________________________________________________________________________

Which learning method(s) best support the development of your clinical competence?

______________________________________________________________________________________________________________________________________________________________________________________________________________________________________________________________________________

Explain how the curriculum content could be integrated so that teaching would form entities based on the patient’s symptoms rather than the dental specialty.

________________________________________________________________________________________________________________________________________________________________________________________________________________________________________________________________________________________________________________________________________________________________________

IV Assessment of each dental specialty

What is the most important/first thing that comes to your mind from each dental specialty that benefits the dental clinical work?

Radiology ________________________________________________________________

Oral and maxillofacial surgery _______________________________________________

Oral pathology ____________________________________________________________

Periodontology ____________________________________________________________

Cariology and endodontics __________________________________________________

Paediatric dentistry ________________________________________________________

Prosthodontics and the function of TMJ _______________________________________

Orthodontics ______________________________________________________________

Dental public health ________________________________________________________

**Table A5:** V How has the learning of the following areas been implemented in the curriculum? What do you think about the following statements?

	Strongly Disagree	Disagree	Neutral	Agree	Strongly Agree
I have developed professionally during my education	1	2	3	4	5
I have sufficient communication and interaction skills to communicate with patients and their loved ones.	1	2	3	4	5
I have sufficient communication and interaction skills to communicate with healthcare professionals.	1	2	3	4	5
I have sufficient knowledge basis, computer skills, and critical thinking to tell the difference between a benign and pathological finding in the mouth.	1	2	3	4	5
I can gather the necessary clinical information about a patient’s general health.	1	2	3	4	5
Without a problem, I can manage diagnostics and treatment planning.	1	2	3	4	5
In my work, I achieve and maintain oral health.	1	2	3	4	5
I am qualified to act as a promoter of oral health for both individuals and large groups.	1	2	3	4	5

**Table A6:** VI Assessment methods. Evaluate how different assessment methods support your learning. Circle the option “Not applicable” if you have not participated in the learning method.

	Not at All Useful	Slightly Useful	Moderately Useful	Very Useful	Extremely Useful	Not Applicable
Entrance examinations	1	2	3	4	5	0
Random tests	1	2	3	4	5	0
Examinations during study courses	1	2	3	4	5	0
Online examination	1	2	3	4	5	0
Course examination	1	2	3	4	5	0
Assessment of final lab work (e.g., during simulations)	1	2	3	4	5	0
Feedback from group work	1	2	3	4	5	0
Written feedback from teachers on clinical work	1	2	3	4	5	0
Oral feedback from teachers on clinical work	1	2	3	4	5	0
Final examinations on each dental specialty	1	2	3	4	5	0
Clinical competency assessments	1	2	3	4	5	0
Peer review	1	2	3	4	5	0
Self-evaluation	1	2	3	4	5	0

VII Self-evaluation.

Give a numeric assessment of your knowledge and skills as a dentist at the moment. _______

1 = Adequate (Clear gaps in skills that need to be improved in order to succeed the dentists’ clinical work)

2 = Satisfactory (Minor deficiencies in skills that need to be improved in order to succeed in dentists’ clinical work)

3 = Good (Competence is at a good level, I can manage dentists’ clinical work)

4 = Commendable (Competence is systematic, I can manage dentists clinical work excellently)

5 = Excellent (Competence is systematic and extensive. I can manage patient care like an experienced dentist)

How has work experience with a dental assistant, dental technician and dental student benefitted you in developing your skills as a dentist? _______

1 = Very poorly

2 = Poorly

3 = Moderately

4 = Well

5 = Very well

0 = Not applicable

Was the teaching given during the autumn of the third study year have had time to be structured and utilized when you started caring for your own patients? _______

1 = Very poorly

2 = Poorly

3 = Moderately

4 = Well

5 = Very well

VIII Other feedback

What other feedback do you want to give about the dental studies?

__________________________________________________________________________________________________________________________________________________________________________________________________________________________________________________________________________________________________________________________________________________________________________________________________________________________________________________________________

IX Ideas on development

What ideas do you have for developing the dental studies?

__________________________________________________________________________________________________________________________________________________________________________________________________________________________________________________________________________________________________________________________________________________________________________________________________________________________________________________________________

X Informed consent

I give permission to use my answers in the research material ☐Yes ☐No
